# Assessment of Racial Disparities in the Risks of Septic and Aseptic Revision Total Knee Replacements

**DOI:** 10.1001/jamanetworkopen.2021.17581

**Published:** 2021-07-21

**Authors:** Anne R. Bass, Huong T. Do, Bella Mehta, Stephen Lyman, Serene Z. Mirza, Michael Parks, Mark Figgie, Lisa A. Mandl, Susan M. Goodman

**Affiliations:** 1 Division of Rheumatology, Hospital for Special Surgery, New York, New York; 2 Division of Rheumatology, Weill Cornell Medicine, New York, New York; 3 Research Administration, Hospital for Special Surgery, New York, New York; 4 Research Division, Hospital for Special Surgery, New York, New York; 5 Department of Population Health Sciences, Weill Cornell Medicine, New York, New York; 6 Medical Education Department, Kyushu University School of Medicine, Fukuoka, Japan; 7 Touro College of Osteopathic Medicine, New York, New York; 8 Department of Orthopedic Surgery, Hospital for Special Surgery, New York, New York; 9 Department of Orthopedic Surgery, Weill Cornell Medicine, New York, New York

## Abstract

**Importance:**

Black patients are at higher risk of revision total knee replacement (TKR) than White patients, but whether racial disparities exist for both septic and aseptic revision TKR and the reason for any disparities are unknown.

**Objective:**

To assess the risk of septic and aseptic revision TKR in Black and White patients and to examine interactions among race and socioeconomic and hospital-related variables that are associated with revision TKR risk.

**Design, Setting, and Participants:**

This cohort study included residents of New York, California, and Florida who underwent TKR. Patient-level data were obtained from the New York Statewide Planning and Research Cooperative System, California’s Office of Statewide Health Planning and Development Patient Discharge Database, and Florida’s Healthcare Utilization Project State Inpatient Database from January 1, 2004, to December 31, 2014. Community characteristics were calculated from the US Census and linked to discharges by patient zip code. American Hospital Association Annual Survey data were linked to discharges using hospital identifiers. The analyses were performed from March 1 to October 30, 2020, with subsequent analyses in April 2021.

**Main Outcomes and Measures:**

Cox proportional hazards regression modeling was used to measure the association of race with septic and aseptic revision TKR.

**Results:**

A total of 722 492 patients underwent primary TKR, of whom 445 616 (61.68%) were female and 61 092 (8.46%) were Black. Black patients were at higher risk of septic (hazard ratio [HR], 1.11; 95% CI, 1.03-1.20) and aseptic (HR, 1.39; 95% CI, 1.33-1.46) revision TKR compared with White patients. Other risk factors for septic revision TKR were diabetes (HR, 1.24; 95% CI, 1.17-1.30), obesity (HR, 1.13; 95% CI, 1.17-1.30), kidney disease (HR, 1.42; 95% CI, 1.29-1.57), chronic obstructive pulmonary disease (HR, 1.22; 95% CI, 1.15-1.30), inflammatory arthritis (HR, 1.53; 95% CI, 1.39-1.69), surgical site complications during the index TKR (HR, 2.19; 95% CI, 1.87-2.56), Medicaid insurance (HR, 1.17; 95% CI, 1.04-1.31), and low annual TKR volume at the hospital where the index TKR was performed (HR, 1.54; 95% CI, 1.41-1.68). Risk factors for aseptic revision TKR were male sex (HR, 1.03; 95% CI, 1.00-1.06), workers’ compensation insurance (HR, 1.61; 95% CI, 1.51-1.72), and low hospital TKR volume (HR, 1.14; 95% CI, 1.07-1.22). Patients with obesity had a lower risk of aseptic TKR revision (HR, 0.81; 95% CI, 0.77-0.84). In an analysis within each category of hospital TKR volume, the HR for aseptic revision among Black vs White patients was 1.20 (95% CI, 1.04-1.37) at very-low-volume hospitals (≤89 TKRs annually) compared with 1.68 (95% CI, 1.48-1.90) at very-high-volume hospitals (≥645 TKRs annually).

**Conclusions and Relevance:**

In this cohort study, Black patients were at significantly higher risk of aseptic revision TKR and, to a lesser extent, septic revision TKR compared with White patients. Racial disparities in aseptic revision risk were greatest at hospitals with very high TKR volumes.

## Key Points

**Question:**

Are there racial disparities in both septic and aseptic revision total knee replacement (TKR) risk?

**Findings:**

In this cohort study of 722 492 patients, Black patients were 1.1 times as likely to undergo septic revision TKR and were 1.4 times as likely to undergo aseptic revision TKR compared with White patients. Racial disparities in aseptic revision TKR risk were greatest at hospitals with a high volume of TKRs.

**Meaning:**

These findings suggest that racial disparities in revision TKR are greater for aseptic than for septic revision and are also associated with characteristics of the hospital where the primary TKR was performed.

## Teaser

This cohort study assesses the risk of septic and aseptic revision total knee replacement in Black and White patients and examines interactions among race and socioeconomic and hospital-related variables associated with revision risk.

## Introduction

Black patients are less likely to undergo total knee replacement (TKR) than White patients^1,2^ and have worse TKR outcomes than White patients, including higher 30-day readmission rates^1,3,4^ and worse pain and function 2 years after surgery.^5^ Black patients also have a higher overall risk of revision TKR than White patients.^6^ Although racial disparities exist in many health domains,^7,8,9^ the reason for these disparities is not always clear; however, one reason could be socioeconomic factors. Black patients are nearly twice as likely as White patients to be insured by Medicaid,^10,11^ and, in the case of orthopedic surgery, poor TKR outcomes have been linked to area-based measures of deprivation.^5,12^ Black patients are also more likely to undergo TKR at low-volume and low-quality hospitals,^2,13^ even bypassing hospitals with a high volume of TKR closer to home.^14,15^ Structural racism may play a role in the hospitals that Black patients choose and the quality of care they receive within those hospitals.^16^

Although few studies have analyzed septic and aseptic revision TKR separately,^17,18,19,20^ these 2 outcomes represent very different mechanisms of TKR failure. Given this fact, we herein hypothesize that racial disparities in revision TKR risk will vary depending on the indication for revision (septic vs aseptic). In addition, we argue that only by analyzing these 2 outcomes separately can we identify the factors contributing to racial disparities in revision TKR risk. The objective of this study was to determine whether racial disparities in revision TKR risk exist for both septic and aseptic revision and to explore interactions between race and socioeconomic and hospital-related variables associated with that risk. We used administrative data from 3 large states that record patient race to provide sufficient power to analyze septic and aseptic revision risk separately.

## Methods

This cohort study followed the Strengthening the Reporting of Observational Studies in Epidemiology (STROBE) guideline for reporting. Request for exemption was approved by the Hospital for Special Surgery institutional review board because the study used deidentified administrative data.

### Study Design and Setting

We used data from 3 administrative discharge data sets: the New York Statewide Planning and Research Cooperative System, California Office of Statewide Health Planning and Development Patient Discharge Database, and Florida Healthcare Utilization Project State Inpatient Database. These states were chosen because they were geographically diverse and had a minimum of 5 years of longitudinal tracking available, allowing us to link a primary TKR admission to a subsequent revision TKR admission in the same patient. Data were included from January 1, 2004, to December 31, 2014, because data were available from all 3 states and only *International Classification of Diseases, Ninth Revision, Clinical Modification* (*ICD-9-CM*) coding was used during this period. Analyses were performed March 1 to October 30, 2020, with additional analyses in April 2021.

### Participants

The eligible cohort was defined as Black or White state residents undergoing a primary TKR (*ICD-9-CM* procedure code 81.54). Race categories were as defined in the administrative data set. Because White patients represent the majority of recipients of TKR, they are the best comparison group to examine disparity in outcomes for Black patients. Other races were excluded because their TKR volume was very small, and we were underpowered to analyze their outcomes. We excluded patients with a diagnosis code indicating a prior knee replacement (*ICD-9-CM* code V43.65) to avoid incorrectly reporting the revision of a prior contralateral primary TKR because *ICD-9-CM* procedure codes do not specify laterality. We also excluded out-of-state residents because subsequent treatment obtained in their home state would not be captured within the database.

### Outcomes

We used *ICD-9-CM* procedure codes to identify patients who underwent revision TKR (eTable 1 in the Supplement). The reason for revision TKR was also determined using *ICD-9-CM* diagnosis codes and categorized as septic, fracture, mechanical (ie, aseptic), or other (eTable 2 in the Supplement). Survival time (time to event) was calculated as the time to first revision. Patients were censored at the time of first revision if they had revision TKR for another cause (eg, in the analysis of aseptic revision, patients were censored if their first revision was a septic revision TKR) or underwent sequential TKR (ie, had TKR of both knees during separate admissions) because the laterality of revision TKR is not available from *ICD-9-CM* procedure codes. Patients who did not experience a revision were censored at time to the end date of the data (December 31, 2014) or the patient’s life expectancy based on annual Centers for Disease Control and Prevention age- and sex-adjusted life tables, whichever was shorter.

### Variables

We included covariates that have been linked to revision TKR risk in the literature,^21^ including age, sex, insurance, comorbidities (ie, diabetes, obesity, kidney disease, and chronic obstructive pulmonary disorder), joint-specific diagnoses (ie, osteoarthritis, osteonecrosis, rheumatoid arthritis, psoriatic arthritis, and spondyloarthropathy), surgical complications during the index TKR (ie, hemorrhage, wound disruption, and retained foreign body), any infection during the index TKR admission, hospital type (ie, nongovernment, not-for-profit, teaching hospital, hospital rural location), bed size, and hospital’s annual TKR volume.

Patient comorbidities, indication for surgery, and surgical site complications or infection during the index TKR were determined using *ICD-9-CM* diagnosis codes (eTable 3 in the Supplement). Two zip code–level community characteristics—percentage of the population under the poverty line (community poverty) and percentage of the population older than 25 years with no college education (community educational level)—were calculated from US Census data and linked by patient zip code to the data sets. Hospital characteristics were obtained from the American Hospital Association Annual Survey to identify hospital-level variables, including teaching status and bed size, for risk adjustment. Hospital TKR volume was calculated as the number of TKR admissions during the discharge year of the patient’s index TKR and categorized as reported by Wilson et al.^22^ We used hospital bed size categories as defined by Dy et al.^21^ All covariates were entered into our final model.

### Statistical Analyses

Continuous variables are summarized as mean (SD) and were compared using 2-tailed, unpaired *t* tests. Categorical variables are summarized as frequency (percentage) and were compared using χ^2^ tests. Kaplan-Meier methods and log-rank tests were used to assess the univariable association between race and septic or aseptic revision TKR. Multivariable Cox proportional hazards regression models were developed to evaluate the association between race and septic or aseptic revision TKR. To examine the possibility of bias introduced by using administrative data from multiple sites, we also analyzed our data using a marginal Cox proportional hazards regression model, a Cox proportional hazards regression model stratified by state, and a Cox proportional hazards regression model with a frailty term. Finally, we examined the interaction between race and community poverty and between race and category of hospital TKR volume, with adjustment for patient, community, and hospital variables. Two-sided *P* < .05 indicated statistical significance. To further explore interactions between race and other variables associated with revision risk, we performed subgroup analyses for the Black and White populations separately. Separate models were also run for subgroups defined by category of hospital annual TKR volume.

## Results

### Baseline Characteristics

This study included 722 492 patients undergoing primary TKR, with a mean (SD) age of 67.3 (10.3) years; 445 616 (61.68%) were female and 276 876 (38.32%) were male, and 61 092 (8.46%) were Black (Table 1 and eFigure 1 in the Supplement). A total of 134 337 patients (18.59%) had diabetes, and 136 183 (18.85%) had obesity. Insurance coverage was Medicare for 432 681 patients (59.89%), Medicaid for 16 381 patients (2.27%), and workers’ compensation for 22 653 patients (3.14%). A total of 5258 patients (0.73%) had a surgical complication coded during their index TKR admission. A total of 144 083 TKR procedures (19.94%) were performed at hospitals with an annual hospital TKR volume of 645 or greater, and 59 643 (8.26%), at hospitals with an annual hospital TKR volume of 89 or less.

**Table 1.** Characteristics of Black and White Patients Undergoing TKR

Characteristic	Patient population^a^		
	Overall (N = 722 492)	White (n = 661 400)	Black (n = 61 092)
Age, mean (SD), y	67.3 (10.3)	67.7 (10.2)	63.3 (10.5)
Sex			
Female	445 616 (61.68)	400 336 (60.53)	45 280 (74.12)
Male	276 876 (38.32)	261 064 (39.47)	15 812 (25.88)
US state			
New York	195 282 (27.03)	173 393 (26.22)	21 889 (35.83)
California	280 190 (38.78)	260 666 (39.41)	19 524 (31.96)
Florida	247 020 (34.19)	227 341 (34.37)	19 679 (32.21)
Insurance			
Medicare	432 681 (59.89)	402 751 (60.89)	29 930 (48.99)
Medicaid	16 381 (2.27)	11 180 (1.69)	5201 (8.51)
Private	236 370 (32.72)	215 564 (32.59)	20 806 (34.06)
Workers’ compensation	22 653 (3.14)	19 416 (2.94)	3237 (5.30)
Other	14 407 (1.99)	12 489 (1.89)	1918 (3.14)
Comorbidities			
Diabetes	134 337 (18.59)	117 096 (17.70)	17 241 (28.22)
Obesity	136 183 (18.85)	118 775 (17.96)	17 408 (28.49)
Kidney disease	28 122 (3.89)	24 378 (3.69)	3744 (6.13)
COPD	92 292 (12.77)	86 917 (13.14)	5375 (8.80)
TKR indication			
Osteoarthritis	702 537 (97.24)	643 568 (97.30)	58 969 (96.52)
Osteonecrosis	4719 (0.65)	4396 (0.66)	323 (0.53)
Dislocation	1178 (0.16)	1077 (0.16)	101 (0.17)
Inflammatory arthritis^b^	23 365 (3.23)	20 620 (3.12)	2745 (4.49)
Index TKR admission			
Surgical complication^c^	5258 (0.73)	4601 (0.70)	657 (1.08)
Any infection	112 211 (15.53)	104 835 (15.85)	7376 (12.07)
US Census community variables, mean (SD), %			
No college	44 (15)	43 (15)	53 (15)
Below poverty level	11 (7)	10 (6)	19 (11)
Hospital annual TKR volume			
≤89	59 643 (8.26)	51 480 (7.78)	8163 (13.36)
90-235	189 688 (26.25)	172 752 (26.12)	16 936 (27.72)
236-644	329 078 (45.55)	302 580 (45.75)	26 498 (43.37)
≥645	144 083 (19.94)	134 588 (20.35)	9495 (15.54)
Hospital bed capacity			
6-199	205 464 (28.44)	193 561 (29.27)	11 903 (19.48)
200-399	290 267 (40.18)	267 353 (40.42)	22 914 (37.51)
≥400	226 761 (31.39)	200 486 (30.31)	26 275 (43.01)
Hospital type			
Government, nonfederal	70 267 (9.73)	63 836 (9.65)	6431 (10.53)
Nongovernment (not-for-profit)	533 384 (73.83)	487 522 (73.71)	45 862 (75.07)
Investor-owned (for-profit)	118 841 (16.45)	110 042 (16.64)	8799 (14.40)
Teaching	279 270 (38.65)	244 347 (36.94)	34 923 (57.16)
Rural	33 358 (4.62)	32 783 (4.96)	575 (0.94)
Indication for revision			
Aseptic (mechanical)	17 563 (2.43)	15 346 (2.32)	2217 (3.63)
Septic (infection)	8830 (1.22)	7863 (1.19)	967 (1.58)
Fracture	823 (0.11)	753 (0.11)	70 (0.11)
Other	346 (0.05)	297 (0.04)	49 (0.08)
Censored	6664 (0.92)	5826 (0.88)	838 (1.37)

Abbreviations: COPD, chronic obstructive pulmonary disease; TKR, total knee replacement.

^a^ Unless indicated otherwise, data are expressed as No. (%) of patients.

^b^ Includes rheumatoid arthritis, psoriatic arthritis, and spondyloarthropathy.

^c^ Includes hemorrhage, wound disruption, and retained foreign body.

Compared with White patients undergoing TKR, Black patients undergoing TKR were younger (mean [SD] age. 63.3 [10.5] vs 67.7 [10.2] years), more likely to be female (45 280 [74.12%] vs 400 336 [60.53%]), and more likely to have diabetes (17 241 [28.22%] vs 117 096 [17.70%]), obesity (17 408 [28.49%] vs 118 775 [17.96%]), kidney disease (3744 [6.13%] vs 24 378 [3.69%]), or inflammatory arthritis (2745 [4.49%] vs 20 620 [3.12%]). Black patients were also more likely to be insured by Medicaid (5201 [8.51%] vs 11 180 [1.69%]) or to have workers’ compensation insurance (3237 [5.30%] vs 19 416 [2.94%]). Black patients’ home communities were characterized by a greater percentage of the population living under the poverty line (mean [SD], 19% [11%] vs 10% [6%]) and lacking any college education (mean [SD], 53% [15%] vs 43% [15%]). Black patients were more likely to have their surgery at a teaching hospital (34 923 [57.16%] vs 244 347 [36.94%]) and at hospitals with 400 or more beds (26 275 [43.01%] vs 200 486 [30.31%]) and less likely to have surgery at a rural hospital (575 [0.94%] vs 32 783 [4.96%]). Black patients were less likely than White patients to undergo TKR at a hospital in which 645 or more TKR procedures were performed annually (9495 [15.54%] vs 134 588 [20.35%]) and more likely to have surgery at a hospital in which 89 or fewer TKR procedures were performed annually (8163 [13.36%] vs 51 480 [7.78%]). The mean (SD) surveillance time in this study was 3.8 (2.8) years.

### Outcomes

**Figure 1.**
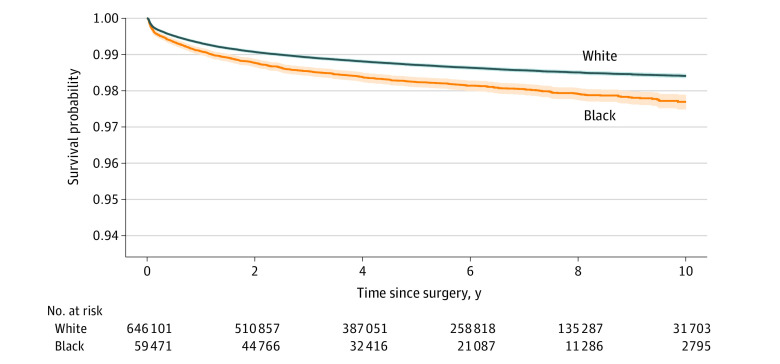
Kaplan-Meier Time-to-Event Analysis Demonstrating Time to Septic Revision Total Knee Replacement in Black vs White Patients Shaded areas indicate 95% CIs.

**Figure 2.**
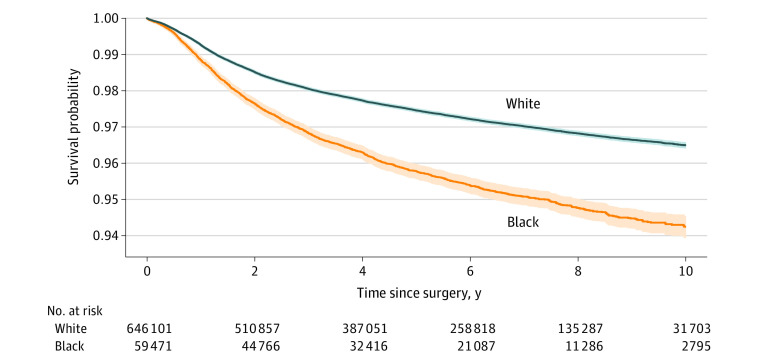
Kaplan-Meier Time-to-Event Analysis Demonstrating Time to Aseptic Revision Total Knee Replacement Revision in Black vs White Patients Shaded areas indicate 95% CIs.

A total of 2217 Black patients (3.63%) and 15 346 White patients (2.32%) underwent aseptic revision TKR, whereas 967 Black patients (1.58%) and 7863 White patients (1.19%) underwent septic revision TKR (Table 1). Among Black patients undergoing revision (n = 3303), 2217 (67.12%) of revisions were aseptic and 967 (29.28%) were septic compared with White patients undergoing revision (n = 24 259) (15 346 [63.26%] and 7863 [32.41%], respectively). The cumulative incident rates for septic revision among Black patients were 0.9% (95% CI, 0.8%-1.0%) at 1 year, 1.5% (95% CI, 1.4%-1.6%) at 3 years, and 1.8% (95% CI, 1.6%-1.9%) at 5 years; among White patients, rates were 0.7% (95% CI, 0.7%-0.7%) at 1 year, 1.1% (95% CI, 1.1%-1.1%) at 3 years, and 1.3% (95% CI, 1.3%-1.3%) at 5 years. Aseptic revision cumulative incident rates among Black patients were 1.1% (95% CI, 1.1%-1.2%) at 1 year, 3.2% (95% CI, 3.0%-3.3%) at 3 years, and 4.2% (95% CI, 4.0%-4.4%) at 5 years; among White patients, rates were 0.7% (95% CI, 0.7%-0.7%) at 1 year, 1.9% (95% CI, 1.9%-2.0%) at 3 years, and 2.5% (95% CI, 2.5%-2.6%) at 5 years. Kaplan-Meier survival curves stratified by race for aseptic and septic revision are presented in Figure 1 and Figure 2.

### Adjusted Models

The Cox multivariable proportional hazards regression model showed that Black patients were at higher risk of both septic revision TKR (hazard ratio [HR], 1.11; 95% CI, 1.03-1.20) and aseptic revision TKR (HR, 1.39; 95% CI, 1.33-1.46) compared with White patients (Table 2). The HRs for the alternative models (marginal Cox proportional hazards regression analysis, Cox proportional hazards regression analysis stratified by state, and a Cox proportional hazards regression model with frailty term) were similar to findings in the primary analysis (eTables 6 and 7 in the Supplement).

**Table 2.** Risk Factors for Aseptic and for Septic TKR Revision: Results of Multivariable Cox Proportional Hazards Regression Analysis

Variable	Aseptic revision		Septic revision	
	HR (95% CI)	*P* value	HR (95% CI	*P* value
Age 5 y	0.78 (0.77-0.79)	<.001	0.85 (0.83-0.86)	<.001
Male sex	1.03 (1.00-1.06)	.046	1.61 (1.55-1.68)	<.001
Black race	1.39 (1.33-1.46)	<.001	1.11 (1.03-1.20)	.004
Insurance				
Medicare	1 [Reference]	NA	1 [Reference]	NA
Medicaid	0.94 (0.86-1.03)	.17	1.17 (1.04-1.31)	.009
Private	0.91 (0.88-0.95)	<.001	0.76 (0.72-0.80)	<.001
Workers’ compensation	1.61 (1.51-1.72)	<.001	0.95 (0.85-1.06)	.33
Other	0.92 (0.83-1.01)	.09	0.93 (0.81-1.07)	.30
Comorbidities				
Diabetes	1.02 (0.98-1.07)	.24	1.24 (1.17-1.30)	<.001
Obesity	0.81 (0.77-0.84)	<.001	1.13 (1.07-1.19)	<.001
Kidney disease	0.86 (0.77-0.95)	.003	1.42 (1.29-1.57)	<.001
COPD	1.05 (0.99-1.10)	.09	1.22 (1.15-1.30)	<.001
Inflammatory arthritis^a^	0.76 (0.70-0.84)	<.001	1.53 (1.39-1.69)	<.001
Surgical complication at index admission^b^	1.15 (0.99-1.34)	.07	2.19 (1.87-2.56)	<.001
US Census community variables				
No college	0.91 (0.81-1.02)	.11	1.14 (0.97-1.35)	.11
Below poverty	0.98 (0.93-1.03)	.48	1.01 (0.94-1.09)	.74
Hospital annual TKR volume				
≥645	1 [Reference]	NA	1 [Reference]	NA
236-644	1.04 (0.98-1.07)	.23	1.13 (1.06-1.21)	<.001
90-235	1.05 (1.00-1.10)	.06	1.33 (1.24-1.42)	<.001
≤89	1.14 (1.07-1.22)	<.001	1.54 (1.41-1.68)	<.001
Hospital bed capacity				
≥400	1 [Reference]	NA	1 [Reference]	NA
200-399	1.04 (1.00-1.08)	.07	0.99 (0.94-1.05)	.75
6-199	1.03 (0.98-1.08)	.22	1.01 (0.94-1.08)	.79
Hospital type				
Nongovernment (not-for-profit)	1 [Reference]	NA	1 [Reference]	NA
Government, nonfederal	1.03 (0.98-1.09)	.21	1.08 (1.00-1.16)	.04
Investor-owned (for-profit)	1.10 (1.05-1.15)	<.001	1.12 (1.05-1.20)	<.001
Rural	0.98 (0.91-1.05)	.51	0.86 (0.77-0.96)	.005
Teaching hospital	0.94 (0.90-0.98)	.001	1.14 (1.08-1.20)	<.001
US state				
New York	1 [Reference]	NA	1 [Reference]	NA
California	1.12 (1.08-1.17)	<.001	1.16 (1.10-1.23)	<.001
Florida	1.06 (1.01-1.11)	.009	0.92 (0.86-0.98)	.008
Calendar year	1.02 (1.01-1.03)	<.001	1.03 (1.02-1.03)	<.001

Abbreviations: COPD, chronic obstructive pulmonary disease; HR, hazard ratio; NA, not applicable; TKR, total knee replacement.

^a^ Includes rheumatoid arthritis, psoriatic arthritis, and spondyloarthropathy.

^b^ Includes hemorrhage, wound disruption, and retained foreign body.

#### Septic Revision TKR

Factors associated with an increased risk of septic revision TKR besides Black race were male sex (HR, 1.61; 95% CI, 1.55-1.68), Medicaid insurance (HR, 1.17; 95% CI, 1.04-1.31), and comorbidities such as diabetes (HR, 1.24; 95% CI, 1.17-1.30), obesity (HR, 1.13; 95% CI, 1.07-1.19), kidney disease (HR, 1.42; 95% CI, 1.29-1.57), and chronic obstructive pulmonary disease (HR, 1.22; 95% CI, 1.15-1.30). The risk of septic revision TKR was also significantly higher in patients with inflammatory arthritis (HR, 1.53; 95% CI, 1.39-1.69), patients who experienced a complication during their index surgery (HR, 2.19; 95% CI, 1.87-2.56), and patients who had their index surgery at a hospital with a very low TKR volume (HR, 1.54; 95% CI, 1.41-1.68). Older age was associated with a lower risk of septic revision TKR (HR, 0.85; 95% CI, 0.83-0.86).

#### Aseptic Revision TKR

Factors associated with an increased risk of aseptic revision TKR besides Black race included male sex (HR, 1.03; 95% CI, 1.00-1.06), workers’ compensation insurance (HR, 1.61; 95% CI, 1.51-1.72), and having the index TKR at a hospital with a very low TKR volume (HR, 1.14; 95% CI, 1.07-1.22) or at an investor-owned (for-profit) hospital (HR, 1.10; 95% CI, 1.05-1.15). Older age (HR, 0.78; 95% CI, 0.77-0.79), as well as some comorbidities, such as obesity (HR, 0.81; 95% CI, 0.77-0.84), kidney disease (HR, 0.86; 95% CI, 0.77-0.95), and inflammatory arthritis (HR, 0.76; 95% CI, 0.70-0.84), were associated with a lower risk of aseptic revision. The risk of aseptic revision TKR was lower in New York than in California (HR, 1.12; 95% CI, 1.08-1.17) and Florida (HR, 1.06; 95% CI, 1.10-1.11).

### Subgroup Analyses

The model including an interaction term between race and category of annual hospital TKR volume showed this interaction to be significant (HR, 1.11; 95% CI, 1.05-1.16). Therefore, we performed additional multivariable models in Black and White patients separately, which yielded similar results except with regard to the association between hospital TKR volume category and aseptic revision TKR risk. Among Black patients, the adjusted risk of aseptic revision TKR was highest for those who underwent their primary TKR at a very-high-volume hospital (≥645 TKRs annually), whereas the analysis for White patients showed aseptic revision TKR risk was lowest at very-high-volume hospitals (eTables 4 and 5 in the Supplement). In separate multivariable models of aseptic revision TKR risk for each hospital TKR volume category, the HR for aseptic revision TKR at low-volume hospitals (≤89 annually) was 1.20 (95% CI, 1.04-1.37) for Black vs White patients, whereas at high-volume hospitals (≥645 annually) it was 1.68 (95% CI, 1.48-1.90) (Table 3).

**Table 3.** Risk of Aseptic Revision TKR in Black vs White Patients: Multivariable Regression Models Within Each Hospital Annual TKR Volume Category

Hospital TKR volume per year	HR (95% CI)	*P* value
≤89	1.20 (1.04-1.37)	.01
90-235	1.37 (1.25-1.49)	<.001
236-644	1.40 (1.30-1.51)	<.001
≥645	1.68 (1.48-1.90)	<.001

Abbreviations: HR, hazard ratio; TKR, total knee replacement.

## Discussion

In this study of 722 492 patients undergoing TKR, we found that Black patients were at significantly higher risk of aseptic revision TKR than White patients (HR, 1.39). The risk of septic revision TKR was modestly higher in Black patients compared with White patients (HR, 1.11), but medical comorbidities and surgical complications at the time of the index TKR were stronger risk factors for septic revision. Undergoing TKR at a hospital with a very low TKR volume (≤89 annually) was an important risk factor for septic revision TKR (HR, 1.54) and had a more modest association with aseptic revision TKR risk (HR 1.14). However, when we performed subgroup analyses in Black and White patients separately, we found a significant interaction between hospital TKR volume and aseptic revision risk. In analyses performed for each category of hospital TKR volume separately, we found that Black patients had a risk of aseptic revision TKR of 1.20 compared with White patients if they underwent their primary TKR at a low-volume hospital, whereas their risk was 1.68 if they underwent TKR at hospital with a very high TKR volume.

We cannot determine from our data whether racial disparities in aseptic revision risk at high-volume hospitals result from greater arthritis severity (and resulting joint deformity) among Black patients at the time of their primary TKR, differences in care delivery during their primary TKR procedure/admission, and/or their having greater access to revision surgery when they need it compared with White patients. In our study, as in others,^13^ Black patients were less likely than White patients to undergo TKR at very-high-volume hospitals (15.54% vs 20.35%), but it is possible that Black patients with more complex knee problems (eg, advanced varus, valgus, or flexion deformities) preferentially sought care at very-high-volume hospitals. In 1 study of patients enrolled in a TKR registry at a very-high-volume hospital,^5^ Black patients had significantly worse pain and function than White patients at the time they underwent TKR. Black patients are more likely than White patients to require manipulation under anesthesia for arthrofibrosis/knee stiffness after TKR,^23^ and chronic pain from arthrofibrosis could, in the worst-case scenario, lead to the need for revision.^24^ The fact that, in our study, there were greater racial disparities in the risk of aseptic revision TKR (an elective procedure) than septic revision TKR (a medical emergency) suggests that a greater desire/ability to proceed with revision surgery could theoretically factor into racial differences in aseptic revision TKR risk.

An alternative hypothesis regarding the interaction between Black race and hospital TKR volume is that structural racism leads to an inequitable distribution of health care services within high-volume hospitals.^16^ In 1 large study,^25^ Black patients were more likely than White patients to have an orthopedic resident participate in their TKR surgery. In the nonorthopedic literature, Black patients have been shown to be significantly more likely to undergo laparoscopic surgery under general rather than epidural anesthesia compared with White patients^26^ and less likely to have nonopioid, multimodal analgesia used when they undergo any surgery.^27^ Implicit bias could potentially also affect the delivery of care to Black patients. However, 1 study of trainee and attending surgeons found that although implicit biases of race and social class were present in most of the clinicians, these biases were not associated with clinical decision making.^28^ A recent study analyzing TKR outcomes in patients enrolled in a single integrated health maintenance organization also found a high risk of aseptic revision TKR in Black patients despite identical insurance coverage in all participants.^20^ That study supports the concept that there are unmeasured factors associated with revision TKR risk in Black patients.

### Strengths and Limitations

A strength of our study is that we studied 3 geographically and racially diverse states that differ in their models of health care delivery. The large size of our cohort and relatively large percentage of Black patients undergoing TKR enabled us to analyze risk factors for septic and aseptic revision TKR separately and to parse out the association between race and these 2 distinct outcomes. By linking files to the American Hospital Association Annual Survey and US Census data, we were also able to include hospital- and community-level variables that can often confound analyses of race and surgical outcomes.

This study has some limitations. Because our analysis was limited to 3 states, our results may not be generalizable. We could not use the national Healthcare Cost and Utilization Project data base, Agency for Healthcare Research and Quality because it lacks both patient race and year-to-year revisit files, making analysis of revision TKR at the individual patient level impossible. An alternative would have been to use the national 5% Medicare sample; however, it only includes patients 65 years or older, which also limits generalizability. Of note, the mean (SD) age of Black patients undergoing TKR in our study was 63.3 (10.5) years. We also lacked information about the types of TKR prostheses used; for example, having a constrained prosthesis is a risk factor for aseptic revision TKR.^29,30^ However, in a prior study, Bass et al^31^ found no difference in use of constrained prostheses between Black and White patients. Finally, although we were able to incorporate hospital TKR volume into our analysis, we had no information about surgeon TKR volume, and low surgeon volume is an important risk factor for TKR revision.^22^ If Black patients are more likely to choose a low-volume surgeon, this factor could have led to an overestimation of the independent association between race and revision TKR risk. Claims data do not report trainee involvement in procedures, so we also could not evaluate this aspect of care delivery.

## Conclusions

In this cohort study, Black patients had a significantly higher risk of aseptic and, to a lesser degree, septic revision TKR compared with White patients. If this elevated risk of aseptic revision TKR was associated with Black patients undergoing TKR with more advanced joint deformity than White patients, then efforts to encourage earlier use of TKR among Black patients might lead to better surgical outcomes. Further studies are needed to analyze care delivery models within hospitals that may contribute to racial disparities in TKR outcomes, particularly at high-volume hospitals. For example, hospitals should ensure that processes of care developed to improve surgical outcomes are being accessed and applied equally across racial and ethnic groups. Studies are also needed to analyze the association between patient race and surgeon choice. Although low surgeon TKR volume is associated with greater revision TKR risk,^22^ patients (particularly Black patients) have been shown in other surgical fields to choose their surgeon based on physician referral,^32^ and few patients have access to information about surgeon volume. Educational resources for patients should be developed that outline factors associated with good TKR outcomes. These educational materials should be available to patients visiting primary care physician offices so they can be guided to make the best decision about TKR timing, hospital, and surgeon choice.

## Notes

Supplement. **eTable 1.** Procedure Codes Used to Identify Revision TKR **eTable 2.** Reasons for Revision TKR: *ICD-9-CM* Codes **eTable 3.** Comorbidities, Indication for TKR, Complications Index TKR: *ICD-9-CM* Codes **eTable 4.** Cox Multivariable Proportional Hazards Regression Modeling of Septic and Aseptic Revision TKR Risk in Black Patients Only **eTable 5.** Cox Multivariable Proportional Hazards Regression Modeling of Septic and Aseptic Revision TKR Risk in White Patients Only **eTable 6.** Marginal Cox Proportional Hazards Regression Model, Cox Proportional Hazards Regression Model Stratified by State, and Cox Proportional Hazards Regression Model With a Frailty Term for Risk of Aseptic Revision TKR **eTable 7.** Marginal Cox Proportional Hazards Regression Model, Cox Proportional Hazards Regression Model Stratified by State, and Cox Proportional Hazards Regression Model With a Frailty Term for Risk of Septic Revision TKR **eFigure.** Flow Diagram Showing Patients Included and Excluded From the Study
