# Supplementary material for: Assessment of Racial Disparities in the Risks of Septic and Aseptic Revision Total Knee Replacements
Source: JAMA Netw Open. 2021 Jul 21;4(7):e2117581. doi: 10.1001/jamanetworkopen.2021.17581 (PMC8295735; doi:10.1001/jamanetworkopen.2021.17581)
Supplement: Supplement. — eTable 1. Procedure Codes Used to Identify Revision TKR eTable 2. Reasons for Revision TKR: ICD-9-CM Codes eTable 3. Comorbidities, Indication for TKR, Complications Index TKR: ICD-9-CM Codes eTable 4. Cox Multivariable Proportional Hazards Regression Modeling of Septic and Aseptic Revision TKR Risk in Black Patients Only eTable 5. Cox Multivariable Proportional Hazards Regression Modeling of Septic and Aseptic Revision TKR Risk in White Patients Only eTable 6. Marginal Cox Proportional Hazards Regression Model, Cox Proportional Hazards Regression Model Stratified by State, and Cox Proportional Hazards Regression Model With a Frailty Term for Risk of Aseptic Revision TKR eTable 7. Marginal Cox Proportional Hazards Regression Model, Cox Proportional Hazards Regression Model Stratified by State, and Cox Proportional Hazards Regression Model With a Frailty Term for Risk of Septic Revision TKR eFigure. Flow Diagram Showing Patients Included and Excluded From the Study [file jamanetwopen-e2117581-s001.pdf]

Supplementary Online Content

Bass AR, Do HT, Mehta B, et al. Assessment of racial disparities in the risks of septic and aseptic revision total knee replacements. *JAMA Netw Open*. 2021;4(7):e2117581. doi:10.1001/jamanetworkopen.2021.17581

eTable 1. Procedure Codes Used to Identify Revision TKR
eTable 2. Reasons for Revision TKR: *ICD-9-CM* Codes
eTable 3. Comorbidities, Indication for TKR, Complications Index TKR: *ICD-9-CM* Codes
eTable 4. Cox Multivariable Proportional Hazards Regression Modeling of Septic and Aseptic Revision TKR Risk in Black Patients Only
eTable 5. Cox Multivariable Proportional Hazards Regression Modeling of Septic and Aseptic Revision TKR Risk in White Patients Only
eTable 6. Marginal Cox Proportional Hazards Regression Model, Cox Proportional Hazards Regression Model Stratified by State, and Cox Proportional Hazards Regression Model With a Frailty Term for Risk of Aseptic Revision TKR
eTable 7. Marginal Cox Proportional Hazards Regression Model, Cox Proportional Hazards Regression Model Stratified by State, and Cox Proportional Hazards Regression Model With a Frailty Term for Risk of Septic Revision TKR
eFigure. Flow Diagram Showing Patients Included and Excluded From the Study

This supplementary material has been provided by the authors to give readers additional information about their work.

© 2021 Bass AR et al. *JAMA Network Open*.

eTable 1. Procedure Codes Used to Identify Revision TKR

**Procedure Codes –used to identify TKR revision**	
**Variable**	**Procedure code**
Revision of knee replacement, total (all components)	00.80
Revision of knee replacement, tibial component	
Replacement of tibial baseplate and tibial insert (liner)	00.81
Revision of knee replacement, femoral component	00.82
Revision of knee replacement, patellar component	00.83
Revision of total knee replacement, tibial insert<br><br>Revision of knee replacement, not otherwise specified<br><br>Removal of knee prosthesis without replacement	00.84<br><br>81.55<br><br>80.06

© 2021 Bass AR et al. *JAMA Network Open*.

eTable 2. Reasons for Revision TKR: *ICD-9-CM* Codes

Reasons for revision	Diagnosis	ICD-9 codes
Mechanical/Aseptic	Osteoarthrosis	715.X0; 715.X5; 715.X6; 715.X8;
Mechanical/Aseptic	Pathological dislocation	718.20; 718.25; 718.26; 718.28; 718.29
Mechanical/Aseptic	Recurrent dislocation of joint	718.30; 718.35; 718.36; 718.38; 718.39
Mechanical/Aseptic	Contracture of joint	718.40; 718.45; 718.46; 718.48; 718.49;
Mechanical/Aseptic	Ankylosis of joint	718.50; 718.55; 718.56; 718.58; 718.59
Mechanical/Aseptic	Other joint derangement not elsewhere classified	718.90; 718.95; 718.96; 718.98; 718.99
Mechanical/Aseptic	Other and unspecified disorders of bone and cartilage	733.90; 733.99
Mechanical/Aseptic	Other acquired deformities of knee	736.6
Mechanical/Aseptic	Mechanical complication of internal orthopedic device implant and graft	996.40-996.43; 996.45-996.49
Mechanical/Aseptic	Mechanical complication due to other implant and internal device, not elsewhere classified	996.59
Mechanical/Aseptic	Other complications due to internal prosthetic device, implant, and graft	996.70; 996.77; 996.78; 996.79
Septic	Pyogenic arthritis<br>Unspecified infective arthritis<br>Osteomyelitis	711.0X<br>711.9X<br>730.0X, 730.1X, 730.2X
Septic	Infection and inflammatory reaction due to internal prosthetic device implant and graft	996.60; 996.66; 996.67; 996.69
Septic	Infected postoperative seroma	998.51
Septic	Other postoperative infection	998.59
Fracture	Pathologic fracture	733.10; 733.14-733.19;
Fracture	Malunion and nonunion of fracture	733.8X;
Fracture	Other and unspecified disorders of bone and cartilage – stress fracture	733.93; 733.96-733.98
Fracture	Fracture of other and unspecified parts of femur	821.X
Fracture	Fracture of patella	822.X
Fracture	Fracture of tibia and fibula	823.X
Fracture	Other multiple and ill-defined fractures of lower limb	827.X

© 2021 Bass AR et al. *JAMA Network Open*.

Reasons for revision	Diagnosis	ICD-9 codes
Other	Disruption of operation wound	998.3X
Other	Foreign body accidentally left during a procedure	998.4
Other	Persistent postoperative fistula	998.6
Other	Acute reaction to foreign substance accidentally left during a procedure	998.7
Other	Non-healing surgical wound	998.83
Other	Unspecified complication of procedure, not elsewhere classified	998.89; 998.9
Other	Revisions lacking any specific codes for infection, mechanical causes or fracture	

ICD-9-CM = International Classification of Diseases, 9th Revision, Clinical Modification.
X = Indicates that the variable includes all codes starting with the numbers preceding the “X”.

© 2021 Bass AR et al. *JAMA Network Open*.

eTable 3. Comorbidities, Indication for TKR, Complications Index TKR: *ICD-9-CM* Codes

Comorbidities	Diagnosis	ICD-9 codes
Diabetes	Diabetes	250.X
Obesity	Obesity	278.0X
Renal disease	Renal disease	403.X; 581.X-583.X; 585.X-587.X
COPD	Chronic obstructive pulmonary disease	491.2X, 492.X

Indication for index TKR	Diagnosis	ICD-9 codes
Osteoarthritis	Osteoarthrosis	715.X0; 715.X5; 715.X6; 715.X8;
Osteonecrosis	Osteonecrosis	733.4X
Inflammatory arthritis	Rheumatoid arthritis	714.0
	Psoriatic arthritis	696.0
	Spondyloarthropathy	720.X; 099.3

Surgical complications index surgery	Diagnosis	ICD-9 codes
Surgical complications		
	Hemorrhage or hematoma complicating a procedure not elsewhere classified	998.1X
	Disruption of operation wound	998.3X
	Foreign body accidentally left during a procedure, Acute reaction to foreign substance accidentally left during a procedure	998.4; 998.7

Medical complications index surgery	Diagnosis	ICD-9 codes
Infection index surgery		
	Other infection due to medical care not elsewhere classified	999.3
	Pneumonia	480-486; 997.3X; 510.X
	Skin infection	035; 680-682; 684; 686.8; 686.9
	Bacterial infection in conditions classified elsewhere and of unspecified site	041.X
	Urinary tract infection, kidney infection	590.X; 597.X; 599.1; 599.9;
	Bacteremia/Sepsis	038.X; 790.7; 995.90; 995.91; 995.92; 998.02
	Upper respiratory tract infection	460-466; 471-476; 034.X
	Gastroenteritis	005.X; 008.X; 009.X;
	Postoperative infection not elsewhere classified	998.5X
	Diverticulitis, peritonitis	562.11; 567.X

© 2021 Bass AR et al. *JAMA Network Open*.

eFigure. Flow Diagram Showing Patients Included and Excluded From the Study

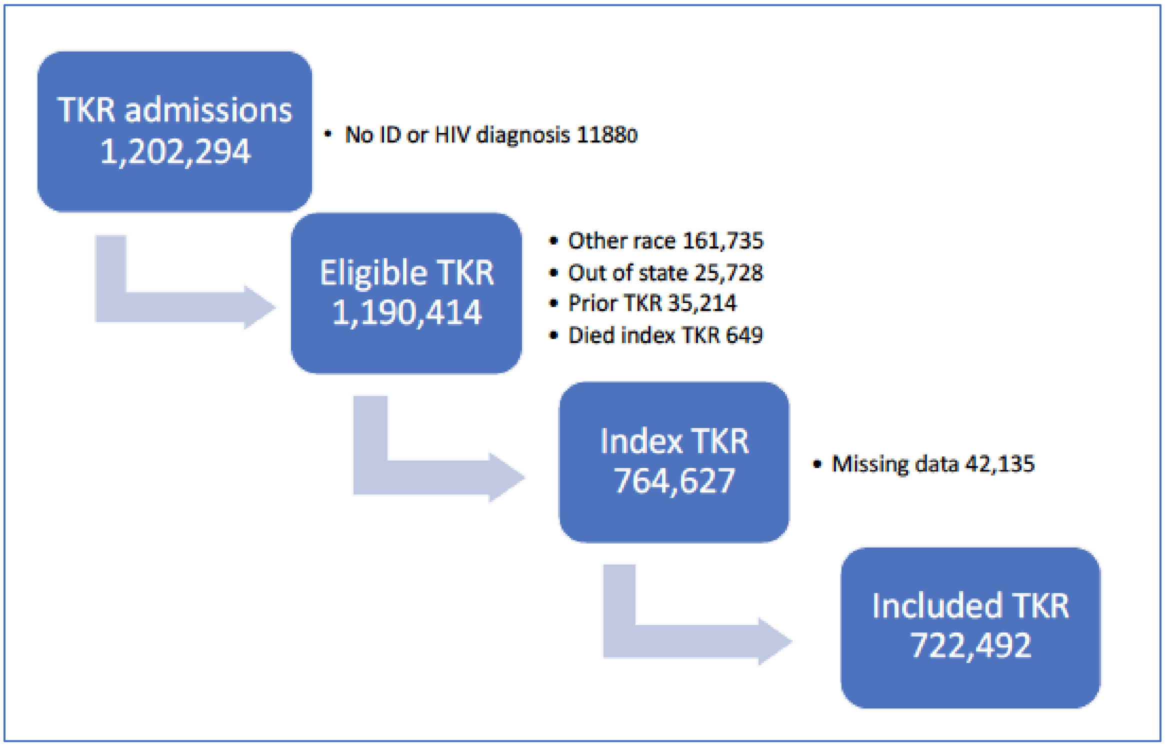

© 2021 Bass AR et al. *JAMA Network Open*.

eTable 4. Cox Multivariable Proportional Hazards Regression Modeling of Septic and Aseptic Revision TKR Risk in Black Patients Only

	Aseptic			Septic		
**Variable**	**HR**	**CI**	**P value**	**HR**	**CI**	**P value**
Age (5yrs)	0.795	(0.78-0.81)	<.0001	0.824	(0.80-0.85)	<.0001
Sex - male	0.977	(0.89-1.08)	0.6296	1.635	(1.43-1.87)	<.0001
**Insurance**						
Medicaid	0.997	(0.85-1.18)	0.9743	0.909	(0.72-1.14)	0.4141
Other	0.830	(0.65-1.07)	0.1454	0.585	(0.40-0.86)	0.0071
Private	0.977	(0.88-1.09)	0.6717	0.676	(0.57-0.80)	<.0001
Workers compensation	1.487	(1.27-1.74)	<.0001	0.779	(0.59-1.03)	0.0797
**Comorbidities**						
Diabetes	1.057	(0.96-1.17)	0.2693	1.150	(1.00-1.33)	0.0555
Obesity	0.895	(0.81-0.99)	0.0254	1.036	(0.90-1.20)	0.6364
Renal disease	0.689	(0.54-0.88)	0.0032	1.393	(1.09-1.78)	0.0086
COPD	1.086	(0.92-1.28)	0.3177	1.206	(0.97-1.50)	0.0883
Inflammatory arthritis*	0.626	(0.49-0.80)	0.0002	1.259	(0.95-1.66)	0.1038
**Index TKR surgery**						
Surgical Complication**	1.333	(0.95-1.86)	0.0919	1.750	(1.15-2.67)	0.0096
**Census tract variables**						
CT % no college	0.822	(0.57-1.18)	0.2846	0.706	(0.41-1.21)	0.2057
CT % poverty	0.969	(0.87-1.08)	0.5647	1.055	(0.90-1.24)	0.5178
**Hospital annual TKR volume** (reference 645+)						
236-644	0.851	(0.75-0.97)	0.0143	1.119	(0.89-1.40)	0.3265
90-235	0.859	(0.75-0.99)	0.0306	1.349	(1.07-1.70)	0.0113
<=89	0.873	(0.74-1.03)	0.1001	1.754	(1.36-2.26)	<.0001
**Hospital bed size**						
200-399 beds	0.961	(0.86-1.07)	0.4636	1.110	(0.95-1.30)	0.2016
6-199 beds	1.066	(0.94-1.21)	0.3213	1.080	(0.88-1.32)	0.4514
**Hospital type**						
Government, non-federal	0.901	(0.78-1.05)	0.1720	1.142	(0.93-1.40)	0.1943
Investor-owned (for-profit)	1.003	(0.87-1.15)	0.9678	1.081	(0.88-1.34)	0.4691
Rural	1.144	(0.78-1.69)	0.4978	1.167	(0.65-2.10)	0.6053
Teaching hospital	0.902	(0.81-1.00)	0.0499	1.242	(1.06-1.46)	0.0077
**State**						
California	1.265	(1.13-1.42)	<.0001	1.256	(1.06-1.49)	0.0080
Florida	1.062	(0.94-1.20)	0.3310	0.951	(0.79-1.14)	0.5936
Year	1.020	(1.00-1.04)	0.0242	1.009	(0.98-1.03)	0.4863

*Rheumatoid arthritis, psoriatic arthritis, spondyloarthropathy
**Hemorrhage, wound disruption, retained foreign body
TKR = Total Knee Replacement

© 2021 Bass AR et al. *JAMA Network Open*.

eTable 5. Cox Multivariable Proportional Hazards Regression Modeling of Septic and Aseptic Revision TKR Risk in White Patients Only

	Aseptic			Septic		
Variable	HR	CI	P value	HR	CI	P value
Age (5yrs)	0.779	(0.77-0.79)	<.0001	0.849	(0.84-0.86)	<.0001
Sex- male	1.041	(1.01-1.08)	0.0177	1.609	(1.54-1.68)	<.0001
**Insurance**						
Medicaid	0.937	(0.84-1.05)	0.2458	1.255	(1.10-1.44)	0.0011
Other	0.942	(0.85-1.05)	0.2821	0.997	(0.86-1.16)	0.9712
Private	0.899	(0.86-0.94)	<.0001	0.773	(0.73-0.82)	<.0001
Workers compensation	1.624	(1.51-1.74)	<.0001	0.978	(0.87-1.10)	0.7040
**Comorbidities**						
Diabetes	1.017	(0.97-1.06)	0.4401	1.247	(1.18-1.32)	<.0001
Obesity	0.785	(0.75-0.82)	<.0001	1.147	(1.08-1.21)	<.0001
Renal disease	0.897	(0.80-1.00)	0.0578	1.426	(1.28-1.59)	<.0001
COPD	1.040	(0.99-1.10)	0.1525	1.221	(1.15-1.30)	<.0001
Inflammatory arthritis	0.789	(0.71-0.87)	<.0001	1.574	(1.42-1.75)	<.0001
**Index TKR surgery**						
Surgical complication	1.112	(0.93-1.32)	0.2313	2.277	(1.92-2.69)	<.0001
**Census tracts**						
CT % no college	0.901	(0.80-1.02)	0.1004	1.186	(1.00-1.41)	0.0543
CT % poverty	0.998	(0.94-1.06)	0.9551	1.021	(0.94-1.11)	0.6277
**Hospital annual TKR volume** (reference 645+)						
236-644	1.052	(1.00-1.10)	0.0354	1.138	(1.06-1.22)	0.0002
90-235	1.079	(1.02-1.14)	0.0041	1.324	(1.23-1.42)	<.0001
<=89	1.204	(1.13-1.29)	<.0001	1.498	(1.36-1.65)	<.0001
**Hospital bed size**						
200-399	1.048	(1.00-1.09)	0.0389	0.973	(0.92-1.03)	0.3799
6-199	1.020	(0.97-1.07)	0.4333	0.997	(0.93-1.07)	0.9320
**Hospital type**						
Government, non-federal	1.060	(1.00-1.12)	0.0348	1.067	(0.99-1.15)	0.0955
Investor-owned (for-profit)	1.118	(1.07-1.17)	<.0001	1.125	(1.05-1.20)	0.0006
rural	0.969	(0.90-1.04)	0.4070	0.854	(0.77-0.95)	0.0042
Teaching hospital	0.944	(0.91-0.98)	0.0050	1.125	(1.06-1.19)	<.0001
**State**						
California	1.103	(1.06-1.15)	<.0001	1.152	(1.08-1.22)	<.0001
Florida	1.058	(1.01-1.11)	0.0209	0.915	(0.86-0.98)	0.0087
Year	1.021	(1.01-1.03)	<.0001	1.028	(1.02-1.04)	<.0001

*Rheumatoid arthritis, psoriatic arthritis, spondyloarthropathy
**Hemorrhage, wound disruption, retained foreign body
TKR = Total Knee Replacement

© 2021 Bass AR et al. *JAMA Network Open*.

eTable 6. Marginal Cox Proportional Hazards Regression Model, Cox Proportional Hazards Regression Model Stratified by State, and Cox Proportional Hazards Regression Model With a Frailty Term for Risk of Aseptic Revision TKR

	Marginal Cox Model			Stratified by State			Frailty Model		
Variable	HR	CI	P value	HR	CI	P value	HR	CI	P value
Age (5yrs)	0.782	(0.77-0.79)	<.0001	0.782	(0.77-0.79)	<.0001	0.782	(0.77-0.79)	<.0001
Race-Black	1.387	(1.36-1.41)	<.0001	1.391	(1.32-1.46)	<.0001	1.391	(1.32-1.46)	<.0001
Sex - Male	1.034	(1.02-1.05)	<.0001	1.032	(1.00-1.06)	0.0455	1.032	(1.00-1.06)	0.0444
**Insurance**									
Medicaid	0.943	(0.89-1.00)	0.0503	0.941	(0.86-1.03)	0.1860	0.939	(0.86-1.03)	0.1691
Other	0.913	(0.82-1.02)	0.1006	0.917	(0.83-1.01)	0.0873	0.917	(0.83-1.01)	0.0885
Private	0.914	(0.88-0.95)	<.0001	0.913	(0.88-0.95)	<.0001	0.913	(0.88-0.95)	<.0001
Workers Compensation	1.629	(1.57-1.69)	<.0001	1.614	(1.51-1.72)	<.0001	1.610	(1.51-1.72)	<.0001
**Comorbidities**									
Diabetes	1.021	(0.96-1.08)	0.4981	1.024	(0.98-1.07)	0.2428	1.024	(0.98-1.07)	0.2410
Obesity	0.809	(0.79-0.83)	<.0001	0.806	(0.77-0.84)	<.0001	0.805	(0.77-0.84)	<.0001
Renal disease	0.859	(0.80-0.92)	<.0001	0.854	(0.77-0.95)	0.0025	0.855	(0.77-0.95)	0.0027
COPD	1.039	(1.00-1.08)	0.0580	1.045	(0.99-1.10)	0.0850	1.045	(0.99-1.10)	0.0884
Inflammatory arthritis*	0.762	(0.64-0.90)	0.0017	0.763	(0.70-0.84)	<.0001	0.763	(0.70-0.84)	<.0001
**Index TKR**									
Surgical Complication**	1.157	(1.05-1.28)	0.0048	1.152	(0.99-1.34)	0.0717	1.152	(0.99-1.34)	0.0722
**Census tracts**									
% no college	0.822	(0.69-0.98)	0.0272	0.910	(0.81-1.02)	0.1130	0.904	(0.80-1.02)	0.0893
% below poverty	1.007	(0.93-1.09)	0.8574	0.981	(0.93-1.03)	0.4787	0.982	(0.93-1.04)	0.5083
**Hospital annual TKR volume**									
236 to 644	1.043	(0.88-1.24)	0.6326	1.025	(0.98-1.07)	0.2746	1.028	(0.98-1.07)	0.2168
90 to 235	1.057	(0.83-1.34)	0.6439	1.046	(1.00-1.10)	0.0697	1.049	(1.00-1.10)	0.0538
<=89	1.153	(0.89-1.50)	0.2869	1.140	(1.07-1.21)	<.0001	1.143	(1.07-1.22)	<.0001
**Hospital bed size**									
200-399	1.055	(0.94-1.19)	0.3657	1.038	(1.00-1.08)	0.0755	1.039	(1.00-1.08)	0.0623
6-199	1.049	(0.92-1.20)	0.4792	1.028	(0.98-1.08)	0.2413	1.030	(0.98-1.08)	0.2135
**Hospital Type**									
Government, non-federal	1.035	(0.94-1.14)	0.4705	1.032	(0.98-1.09)	0.2270	1.034	(0.98-1.09)	0.1977
Investor-owned (for-profit)	1.090	(1.05-1.14)	<.0001	1.101	(1.05-1.15)	<.0001	1.102	(1.05-1.15)	<.0001
Rural	0.968	(0.93-1.01)	0.1648	0.975	(0.91-1.05)	0.4996	0.975	(0.91-1.05)	0.4931
Teaching hospital	0.932	(0.89-0.98)	0.0033	0.938	(0.90-0.97)	0.0008	0.938	(0.90-0.97)	0.0008
**Calendar year**	1.021	(0.99-1.05)	0.1341	1.021	(1.01-1.03)	<.0001	1.021	(1.01-1.03)	<.0001

*Rheumatoid arthritis, psoriatic arthritis, spondyloarthropathy; **Hemorrhage, wound disruption, retained foreign body
TKR = Total Knee Replacement

© 2021 Bass AR et al. *JAMA Network Open*.

eTable 7. Marginal Cox Proportional Hazards Regression Model, Cox Proportional Hazards Regression Model Stratified by State, and Cox Proportional Hazards Regression Model With a Frailty Term for Risk of Septic Revision TKR

	Marginal Cox Model			Stratified by State			Frailty		
Variable	HR	CI	P value	HR	CI	P value	HR	CI	P value
Age (5yrs)	0.845	(0.83-0.86)	<.0001	0.845	(0.83-0.86)	<.0001	0.845	(0.83-0.86)	<.0001
Race-Black	1.105	(1.05-1.17)	0.0004	1.113	(1.03-1.20)	0.0041	1.113	(1.03-1.20)	0.0041
Sex - Male	1.619	(1.53-1.71)	<.0001	1.614	(1.55-1.68)	<.0001	1.613	(1.55-1.68)	<.0001
**Insurance**									
Medicaid	1.176	(1.14-1.22)	<.0001	1.168	(1.04-1.31)	0.0086	1.167	(1.04-1.31)	0.0090
Other	0.920	(0.88-0.97)	0.0009	0.929	(0.81-1.07)	0.2974	0.929	(0.81-1.07)	0.2985
Private	0.761	(0.69-0.84)	<.0001	0.759	(0.72-0.80)	<.0001	0.758	(0.72-0.80)	<.0001
Workers Compensation	0.972	(0.89-1.07)	0.5484	0.950	(0.85-1.06)	0.3495	0.950	(0.85-1.06)	0.3416
**Comorbidities**									
Diabetes	1.227	(1.20-1.25)	<.0001	1.235	(1.17-1.30)	<.0001	1.236	(1.17-1.30)	<.0001
Obesity	1.145	(1.10-1.19)	<.0001	1.132	(1.07-1.19)	<.0001	1.132	(1.07-1.19)	<.0001
Renal disease	1.440	(1.38-1.50)	<.0001	1.424	(1.29-1.57)	<.0001	1.425	(1.29-1.57)	<.0001
COPD	1.207	(1.16-1.26)	<.0001	1.222	(1.15-1.30)	<.0001	1.221	(1.15-1.30)	<.0001
Inflammatory arthritis*	1.526	(1.38-1.69)	<.0001	1.531	(1.39-1.69)	<.0001	1.530	(1.39-1.69)	<.0001
**Index TKR**									
Surgical Complication**	2.212	(1.89-2.60)	<.0001	2.191	(1.87-2.56)	<.0001	2.191	(1.87-2.56)	<.0001
**Census tracts**									
% no college	0.917	(0.82-1.02)	0.1072	1.145	(0.97-1.35)	0.1081	1.136	(0.96-1.34)	0.1297
% below poverty	1.074	(1.04-1.10)	<.0001	1.012	(0.94-1.09)	0.7450	1.014	(0.94-1.09)	0.7025
**Hospital annual TKR volume**									
236 to 644	1.177	(1.01-1.37)	0.0351	1.132	(1.06-1.21)	0.0001	1.135	(1.06-1.21)	0.0001
90 to 235	1.356	(1.19-1.55)	<.0001	1.322	(1.23-1.42)	<.0001	1.327	(1.24-1.42)	<.0001
<=89	1.576	(1.44-1.72)	<.0001	1.535	(1.41-1.68)	<.0001	1.540	(1.41-1.68)	<.0001
**Hospital bed size**									
200-399	1.029	(0.96-1.10)	0.3949	0.990	(0.94-1.05)	0.7316	0.992	(0.94-1.05)	0.7849
6-199	1.058	(1.00-1.12)	0.0688	1.009	(0.94-1.08)	0.7970	1.011	(0.95-1.08)	0.7488
**Hospital Type**									
Government, non-federal	1.080	(0.95-1.23)	0.2421	1.076	(1.00-1.15)	0.0437	1.077	(1.00-1.16)	0.0407
Investor-owned (for-profit)	1.093	(1.03-1.16)	0.0022	1.121	(1.05-1.20)	0.0005	1.120	(1.05-1.19)	0.0005
Rural	0.842	(0.83-0.85)	<.0001	0.860	(0.77-0.96)	0.0052	0.859	(0.77-0.96)	0.0050
Teaching hospital	1.125	(1.04-1.22)	0.0046	1.138	(1.08-1.20)	<.0001	1.140	(1.08-1.20)	<.0001
**Calendar year**	1.025	(1.00-1.05)	0.0152	1.026	(1.02-1.03)	<.0001	1.026	(1.02-1.03)	<.0001

*Rheumatoid arthritis, psoriatic arthritis, spondyloarthropathy; **Hemorrhage, wound disruption, retained foreign body
TKR = Total Knee Replacement

© 2021 Bass AR et al. *JAMA Network Open*.
